# Molecular mechanisms underlying fetal bovine serum-induced developmental delay in mouse embryos: a data-independent acquisition (DIA)-based proteomics study

**DOI:** 10.3389/fmolb.2026.1781512

**Published:** 2026-07-07

**Authors:** Songjie Lv, Chen Ying, Bian Lou, Luhan Zhang, Jiayan Lv, Jia Zhou, Hui Pan

**Affiliations:** 1 Department of Assisted Reproduction, Urumqi Maternal and Child Health Hospital, Urumqi, China; 2 Biotechnology Research Institute, Xinjiang Academy of Animal Sciences, Urumqi, Xinjiang, China; 3 Scientific Research Department, Urumqi Maternal and Child Health Hospital, Urumqi, Xinjiang, China

**Keywords:** data-independent acquisition (DIA) proteomics, embryonic developmental delay, fetal bovine serum (FBS), lipid metabolism, maternal-zygotic transition reactive oxygen species (ROS)

## Abstract

Embryonic developmental delay constitutes a key pathological feature in implantation failure and the early loss of pregnancy in human assisted reproductive technology (ART). This condition is mechanistically linked to mitochondrial dysfunction and epigenetic reprogramming defects during maternal-embryonic communication. Here, we established a mouse model of fetal bovine serum (FBS)-induced embryonic developmental delay and performed comprehensive proteomic profiling with data-independent acquisition (DIA) quantitative proteomics. In addition, we also used immunofluorescence, reactive oxygen species (ROS) determination, and protein inhibitors to determine the specific role of FABP4, a fatty acid transport protein, in the early stages of embryo development. Our analysis identified 165 differentially expressed proteins (DEPs); of these, 103 were significantly downregulated (P < 0.05; fold change >1.5) and 62 were upregulated (P < 0.05; fold change <0.66). Gene ontology and KEGG pathway analyses revealed significant enrichment of DEPs in lipid metabolic reprogramming (particularly arginine-proline metabolism), cytoskeletal organisation, and the negative regulation of RNA splicing. Integrated multi-omics analysis revealed a tripartite regulatory mechanism: (1) cytoskeletal destabilisation, as evidenced by the downregulation of 19/24 cytoskeletal organisation proteins (P < 0.01; false discovery rate [FDR]: 0.089); (2) disrupted nucleocytoplasmic transport and splicing, characterised by the reduced expression of 5/7 ribosomal shuttle proteins and impaired nucleoporin (Kpna2/Kpnb1) functionality; and (3) metabolic dysregulation, resulting in the complete downregulation of all six KEGG-enriched metabolic proteins and PF00061 domain proteins (P < 0.01; FDR: 0.004), including three fatty acid transporters and two fatty acid-binding proteins, indicating compromised ATP synthesis and lipid metabolism. At a concentration of 200 nm, the FABP4 inhibitor BMS-309403 can cause embryonic retardation and increase the levels of ROS. Collectively, our findings demonstrate that the fatty acid transporter FABP4 is expressed in the early 8-cell embryos of mice. We provide new insights into the mechanisms underlying embryonic development delay caused by FBS. Furthermore, proteomic analysis revealed that FABP4 is regulated by an integrated cytoskeleton-nuclear-cytoplasmic transport-metabolic network. The inhibition of FABP4 led to the retardation of embryonic development and increased the levels of ROS.

## 1 Introduction

Embryonic development is a precisely regulated biological process that is governed by spatiotemporally defined molecular networks (Oligny, 2001) and genetically encoded metabolic programmes (Gerri et al., 2020). Mice serve as a well-established model organism for the investigation of mammalian development (Chrysanthou et al., 2022); thus, a mouse model of embryonic developmental delay could provide crucial mechanistic insights into infertility and pregnancy-related disorders. Fetal bovine serum (FBS) is widely employed in embryonic development research to support *in vitro* co-culture systems for embryos and somatic cells (Saadeldin et al., 2014), supplying essential nutrients for cellular proliferation (Saadeldin et al., 2014). Our research team previously developed a mouse model of embryonic developmental delay using FBS. However, given the scarcity of human embryos (typically 10–15 per cycle), the compositional complexity of FBS (Vis et al., 2020), and the potential for dysregulation of multiple molecules to induce developmental abnormalities (Watanabe et al., 2002; Zhao et al., 2024; Ancelin et al., 2016; Zhu et al., 2023; Kalous and Aleshkina, 2023), conventional proteomic methods often prove inadequate for comprehensive analysis. To address these limitations, in this study, we employed data-independent acquisition (DIA) proteomics, a technique that provides enhanced robustness and reduced levels of data loss when compared to data-dependent acquisition (DDA) (Demichev et al., 2022). Crucially, DIA facilitates the precise quantification of low-abundance proteins, including transcription factors and signalling molecules (Martano et al., 2025), thereby enabling a paradigm shift in embryonic research by bridging phenotypic alterations with molecular signatures.

In this study, we combined DIA-based proteomics with multi-tiered functional validation to systematically investigate multi-dimensional regulatory mechanisms. Metabolically, exogenous components disrupt lipid metabolism and amino acid homeostasis (Dunning et al., 2010). The downregulation of fatty acid-binding protein 3 (FABP3) reduces the efficiency of fatty acid uptake, diverting metabolic flux towards the less efficient glycolytic pathway (McKeegan and Sturmey, 2011). Simultaneously, dysregulation of the arginine-proline metabolic axis impairs nitric oxide (NO)-mediated signalling (Bodis et al., 2022), culminating in an embryonic energy deficit. Furthermore, aberrant expression of the nuclear transport protein Kpna2 disrupts nucleocytoplasmic trafficking (Shi. et al., 2023a), while the coordinated downregulation of small nuclear ribonucleoproteins (snRNPs) (Patel and Bellini, 2008) and ribosome assembly factors (Klinge and Woolford, 2019) exacerbates translational inefficiency and developmental asynchrony.

Collectively, our findings delineate the molecular basis of FBS-induced embryonic developmental delay and provide a theoretical framework for understanding human embryonic developmental regulation.

## 2 Materials and methods

### 2.1 Establishment of the animal model

#### 2.1.1 Experimental animal standardisation

Sexually mature female C57BL/6 mice (Animal Experiment Center of Xinjiang Medical University), aged 6–8 weeks and weighing 18–25 g, were housed under specific pathogen-free (SPF) conditions with controlled temperature (22 °C ± 1 °C), humidity (50% ± 5%) and a 12 h light/12 h dark cycle. A total of ten female mice were utilized: seven for model replication and validation (to produce 150 two-cell embryos which were randomly assigned to two groups), and two females for experimental analysis (to produce 50 two-cell embryos which were randomly assigned to two groups). DIA protein sequencing was conducted with three replicates per group, with each replicate including eight embryos. The animal research was conducted in accordance with local experimental guidelines and was approved by the Institutional Animal Ethics Committee of Urumqi Maternal and Child Health Hospital (Reference: XJFYLL2022025).

#### 2.1.2 Synchronisation of oestrous cycles

Vaginal smears were collected daily between 09:00 and 10:00 for five consecutive days. Mice exhibiting characteristics of the diestrus phase (predominantly leukocytes with few epithelial cells) were selected for subsequent procedures.

#### 2.1.3 Superovulation protocol

At 15:00 on the first day of diestrus, mice received an intraperitoneal injection of 5–10 IU of pregnant mare serum gonadotropin (PMSG; dissolved in 0.1 mL sterile saline) to mimic the effects of follicle-stimulating hormone (FSH). Forty-eight hours after the injection of PMSG (on the third day at 15:00), mice were injected intraperitoneally with 5–10 IU of human chorionic gonadotropin (hCG) to simulate the luteinising hormone (LH) surge and induce ovulation.

#### 2.1.4 Mating and the assessment of vaginal plugs

Immediately after the administration of hCG, females were housed with male mice in a 1:2 (male:female) ratio. At 09:00 the next morning, we checked all females for vaginal plugs; successful mating was confirmed by the presence of a white keratinised copulatory plug in the vagina.

#### 2.1.5 Ovulation site collection and processing

At 42–44 h post-hCG injection, mice were anaesthetised with sodium pentobarbital (50 mg/kg) by intraperitoneal injection followed by cervical dislocation for euthanasia. The abdominal cavity was then opened approximately 1.5 cm above the pubic symphysis to allow for the prompt isolation of both oviductal ampullae which were then transferred to pre-warmed G-MOPS medium (Vitrolife, Cat. No. 510131). Next, we used a stereomicroscope (×20 magnification) to mechanically rupture the ampullae to release two-cell embryos. The number of two-cell embryos was recorded (ovulation normally yields 8–12 embryos per mouse; superovulation increases this to 20–30 embryos per mouse).

### 2.2 Intervention of embryo development arrest

#### 2.2.1 Experimental groups and culture conditions

Fetal bovine serum was heated in a water bath at 56 °C for 30 min before being placed in a refrigerator at 4 °C to await further use. Collected embryos were then equally divided into two groups: (1) a Treatment Group in which early fertilised embryos (zygote stage) were transferred to G-1 culture medium (Vitrolife, Cat. No. 510112) containing 10% FBS (Gibco, Cat. No. 10270) and cultured until the target time point, and (2) the Control Group in which embryos were cultured in basal G-1 medium without FBS supplementation for synchronisation.

#### 2.2.2 Collection of embryos

For the normal development embryo group (C-8C): after 24 h of control group culture, we selected embryos that had reached the 8-cell stage (Stage: E2.5) exhibiting normal morphology (homogeneous cells and a fragmentation rate <10%). For the slow development embryo group (M-8C), following 48 h of treatment group culture, we collected 8-cell stage embryos (homogeneous cells and a fragmentation rate <10%).

After 24 h of culture, the different stages of embryonic development (4-cell stage, 8-cell stage, etc.) and the number of cells per embryo were recorded. Using each mouse as an independent biological replicate (n ≥ 6), comparisons between the two groups were performed using a paired t-test. Data are presented as mean ± standard error of the mean (SEM) and P < 0.05 was considered statistically significant.

#### 2.2.3 Embryo handling and storage

For cryoprotectant loading, embryos were transferred to the bottom of 0.6 mL low-binding centrifuge tubes (Eppendorf, Cat. No. 22431021) containing 3 μL of embryo cryopreservation solution (TMED, Kitazato, Cat. No. CT-1123). Eight embryos were transferred per group. For cryopreservation, embryos were flash-frozen in liquid nitrogen and subsequently stored at −80 °C (Thermo Scientific, Model: 902-ULT) for long-term preservation.

### 2.3 DIA detection and data analysis

#### 2.3.1 Sample preparation

SDT buffer (4% sodium dodecyl sulfate (SDS), 100 mM Tris-HCl, pH 7.6) was added to each sample of protein solution. For cell samples, SDC buffer (5% sodium deoxycholate (SDC), 100 mM Tris-HCl, pH 8.5) was used instead. Cell lysates were subject to ultrasonication and then boiled for 15 min, while protein samples were boiled without ultrasonication. Both sets of samples were then centrifuged at 14,000 × g for 40 min; the concentration of the supernatant was then determined with a BCA Protein Assay Kit. Next, 15 µg of each protein sample was then mixed with five-fold the volume of loading buffer and boiled for 5 min. Proteins were then separated by 4%–20% sodium dodecyl sulfate-polyacrylamide gel electrophoresis (SDS-PAGE) under constant voltage (180 V, 45 min). Protein bands were visualized by staining with Coomassie Brilliant Blue R-250.

For serum/plasma, samples were centrifuged at 14,000 × g for 20 min. The concentration of the supernatant was then quantified with the BCA Protein Assay Kit. Next, 15 μg of protein per sample was mixed with five-fold the volume of loading buffer and boiled for 5 min. Proteins were then separated on 4%–20% SDS-PAGE (180 V, 45 min) and stained with Coomassie Brilliant Blue R-250.

In this study, equal amounts from each sample were pooled to form a quality control (QC) sample for subsequent analysis.

#### 2.3.2 Sample digestion

Each sample was treated with dithiothreitol (DTT) and incubated at 37 °C for 1.5 h to reduce disulfide bonds. Subsequently, iodoacetamide (IAA) was added to block the reduced cysteine residues, and the reaction was allowed to proceed in the dark at room temperature for 30 min. Trypsin was then added at a trypsin-to-protein mass ratio of 1:50, and the mixture was incubated overnight at 37 °C (15–18 h). The resulting peptides were desalted using an MCX SPE column (Omicsolution, OS-MCX-1 mL), concentrated under vacuum, and resuspended in 20 μL of aqueous solution containing 0.1% (v/v) formic acid. Peptide concentration was estimated based on ultra-violet (UV) absorbance at 280 nm. For DIA experiments, retention time calibration peptides (iRT) were added to each sample prior to LC-MS/MS analysis.

#### 2.3.3 Mass spectrometry analysis by DIA

Peptides from each sample were analysed in DIA mode using a timsTOF HT mass spectrometer (Bruker Daltonics) coupled with a nanoElute system (Bruker Daltonics). Full-scan MS spectra (MS1) were acquired over a m/z range of 300–1,500. For MS2, the dia-PASEF (parallel accumulation serial fragmentation) acquisition method was employed. In the DIA scan mode, 66 isolation windows were defined, with an accumulation time of 50 milliseconds per window. Under dia-PASEF conditions, the collision energy was linearly correlated with the inverse of ion mobility (1/K0), ranging from 20 to 59 eV, corresponding to ion mobilities between 0.6 and 1.6 Vs/cm^2^.

#### 2.3.4 Mass spectrometry data analysis

DIA data were processed using DIA-NN 2.0 software. Key software parameters were implemented as follows: enzyme specificity was set to trypsin, with a maximum of one missed cleavage allowed; carbamidomethylation of cysteine (C) was selected as a fixed modification, while oxidation of methionine (M) and acetylation of the N-terminus of proteins were selected as variable modifications. All reported results were based on protein identification confidence levels reaching 99%, determined by a false discovery rate (FDR) ≤ 1%. Label-free quantification (LFQ) was employed for quantitation, and median centering was performed to eliminate systematic bias. Differentially expressed proteins were identified by t-tests or by analysis of variance (ANOVA), with multiple testing correction via the Benjamini–Hochberg (BH) method. Proteins with |log2FC| ≥ 1.5 and adjusted P-value (FDR) < 0.05 were considered to be significantly differentially expressed. Data have been uploaded to the Iprox database (https://www.iprox.cn/), with the reference number: IPX0015024000.

### 2.4 Bioinformatics analysis

#### 2.4.1 Clustering analysis

Hierarchical clustering analysis was conducted using Cluster 3.0 (http://bonsai.hgc.jp/∼mdehoon/software/cluster/software.htm) and Java Treeview (http://jtreeview.sourceforge.net). This analysis employed Euclidean distance as the similarity metric and applied average linkage clustering based on the centroid of observations for grouping.

#### 2.4.2 Subcellular localisation prediction

Protein subcellular localisation was predicted using CELLO (http://cello.life.nctu.edu.tw/), a multi-class support vector machine-based classification system.

#### 2.4.3 Domain annotation

Protein domain identification was performed by querying protein sequences against the Pfam database within the InterPro member databases, utilizing the InterProScan software suite.

#### 2.4.4 Gene ontology (GO) annotation

GO annotation was carried out through local homology searches of differentially expressed protein sequences using the NCBI BLAST + client (ncbi-blast-2.2.28+-win32.exe) and InterProScan. Homologous sequences were then mapped to GO terms and annotated using the Blast2GO software package. The GO annotation results were visualized using a custom R script.

#### 2.4.5 Kyoto encyclopaedia of genes and genomes (KEGG) pathway analysis

Following annotation, the proteins of interest were aligned against the KEGG database (https://www.genome.jp/kegg/) to obtain KEGG orthology identifiers. These identifiers were subsequently mapped to known KEGG pathways for functional pathway enrichment analysis.

### 2.5 Detection of FABP4 protein in early embryos

Embryos were fixed with 4% paraformaldehyde in PBS at room temperature. Permeabilization was carried out using 0.5% Triton X-100 in PBS, followed by blocking with 5% BSA in PBS for 1 h at room temperature. Embryos were then incubated overnight with a primary antibody: Alexa Fluor® 647 Anti-FABP4 (Abcam, Cambridge, United Kingdom). Then, incubation with Hoechst 33342 for 10 min was performed to visualize the protein in the dark. Embryos were observed using an LSM880 laser scanning confocal microscope (META, Zeiss, Jena, Germany).

### 2.6 Inhibition of the FABP4 protein

Embryos were divided into two groups, with one group treated with G-1 diluted BMS-309403 (BMS, Princeton, United States) to 200 nm, while the other group received G-1 only. Two-cell embryos were cultured for 2 days, and the developmental progression of the embryos was observed. BMS-309403 has Ki values of <2 nM for FABP4, 250 nM for FABP3, and 350 nM for FABP5; at a concentration of 200 nM, BMS-309403 can completely inhibit FABP4 while preserving the compensatory mechanism of FABP3.

### 2.7 ROS measurement

Embryos were incubated in a 10 µM solution of DCFH-DA (Beyotime Biotechnology, Jiangsu, China) at 37 °C for 30 min. Subsequently, the embryos were washed three times in PBS with 0.1% PVP and placed on a glass slide. Images were then acquired with a fluorescence microscope (Nikon, Tokyo, Japan). The mass spectrometry proteomics data have been deposited to the ProteomeXchange Consortium (https://proteomecentral.proteomexchange.org) via the iProX partner repository [Ma et al., 2019; Chen et al., 2021] with the dataset identifier PXD079804.

## 3 Results

### 3.1 Embryonic development

**FIGURE 1:**
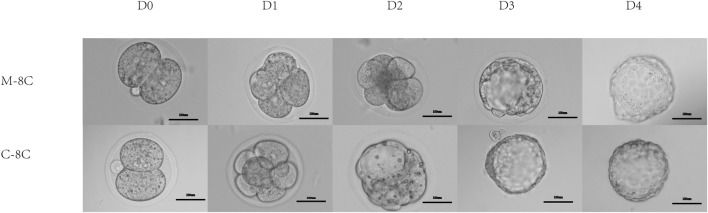
The developmental progression of both the C-8C and M-8C groups from Day 0 (D0, immediately following the collection of two-cell embryos) through Days 1–4 (D1–D4), with microscopy images displayed at specified magnifications.

All seven embryos used for model validation exhibited consistent developmental delays. The normally developing embryo group (C-8C) progressed according to the expected developmental timeline, while the delayed development group (M-8C) exhibited a 24-h lag beginning on Day 1 (D1). Notably, despite this delay, M-8C embryos successfully expanded to form blastocysts and underwent hatching, thus confirming the validity of the established model (Figure 1; Table 1).

**TABLE 1:** Effect of fetal bovine serum (FBS) on the mean cell number per embryo in mouse early embryos.

Mouse	C-8C	M-8C	P-value
1	7.9 ± 0.03	3.8 ± 0.04	1.40312E − 09
2	8 ± 0	3.7 ± 0.05	1.35022E − 09
3	7.9 ± 0.03	3.9 ± 0.03	1.0427E − 05
4	8 ± 0	3.9 ± 0.03	4.20048E − 05
5	8 ± 0	3.8 ± 0.04	0.000187588
6	8 ± 0	4 ± 0	0.000788573
7	8 ± 0	4 ± 0	0.003095116

Data are presented as mean ± SEM., Data were compared statistically using the paired t-test. *P < 0.05 vs. control.

### 3.2 Differential protein analysis between development groups

**FIGURE 2:**
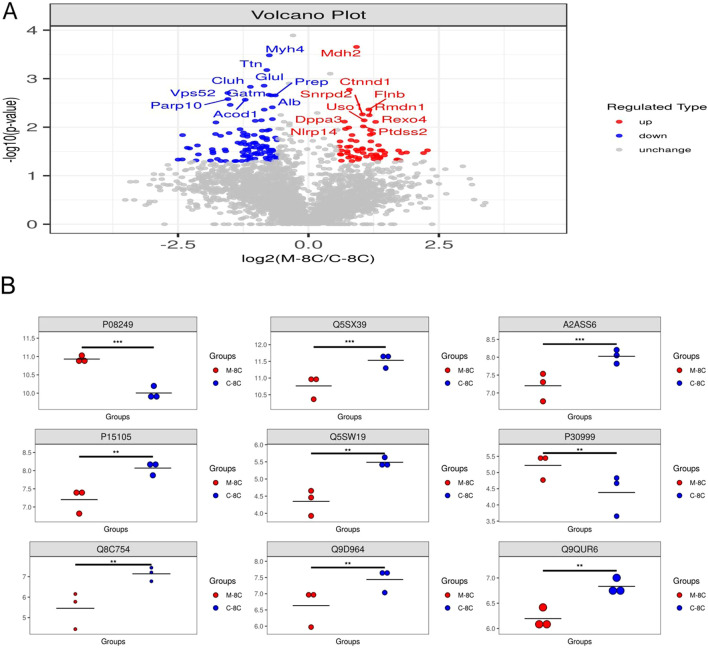
**(A)** Volcano plot showing differentially expressed proteins between normally developing and developmentally delayed embryos. The volcano plot compares protein expression profiles between the normally developing group (C-8C) and the delayed development group (M-8C). The x-axis displays the log_2_-transformed fold change in protein expression, while the y-axis shows the negative log_10_-transformed P-values representing statistical significance. Red data points indicate significantly upregulated proteins, blue points represent significantly downregulated proteins, and grey points denote proteins without significant differential expression. The plot highlights the top 10 most significantly altered proteins (both upregulated and downregulated) with their respective protein IDs. **(B)** Box plots illustrating the expression levels of differentially expressed proteins between normally developing and developmentally delayed embryos. The box plots present comparative protein expression patterns between the normally developing group (C-8C) and the delayed development group (M-8C). The x-axis specifies the experimental groups, while the y-axis represents log_2_-transformed protein expression levels. Red and blue colour coding distinguishes the expression profiles of individual differentially expressed proteins across samples. Statistical significance is indicated by asterisks: *p < 0.001, * 0.001 ≤ p < 0.01, and *0.01 ≤ p < 0.05.

Univariate analysis identified differential proteins between experimental groups using a fold change (FC) threshold >1.5 and P-value <0.05 (t-test). Of the 2,991 proteins detected in total, 165 differentially expressed proteins (DEPs) were identified, including 62 upregulated and 103 downregulated proteins. Of the upregulated proteins, five were associated with mitochondrial metabolism and energy homeostasis: enoyl-CoA delta isomerase (Eci1), fumarate hydratase (Fh), monofunctional C1-tetrahydrofolate synthase (Mthfd1l), mitochondrial import inner membrane translocase subunit Tim9 (Timm9), and NADH dehydrogenase (ubiquinone) iron-sulfur protein 7 (Ndufs7). Additional DEPs included three cytoskeletal structural proteins, two proteins involved in protein synthesis and processing, four regulators of cell cycle, proliferation and signal transduction, four components of intracellular transport and membrane trafficking, and three transcriptional and epigenetic modifiers. Expression patterns were visualized through a volcano plot comparing normal vs. delayed development groups (Figure 2A). Box plots were used to further illustrate inter-group differences in protein expression (Figure 2B).

### 3.3 Bioinformatics analysis of DEPs

**FIGURE 3:**
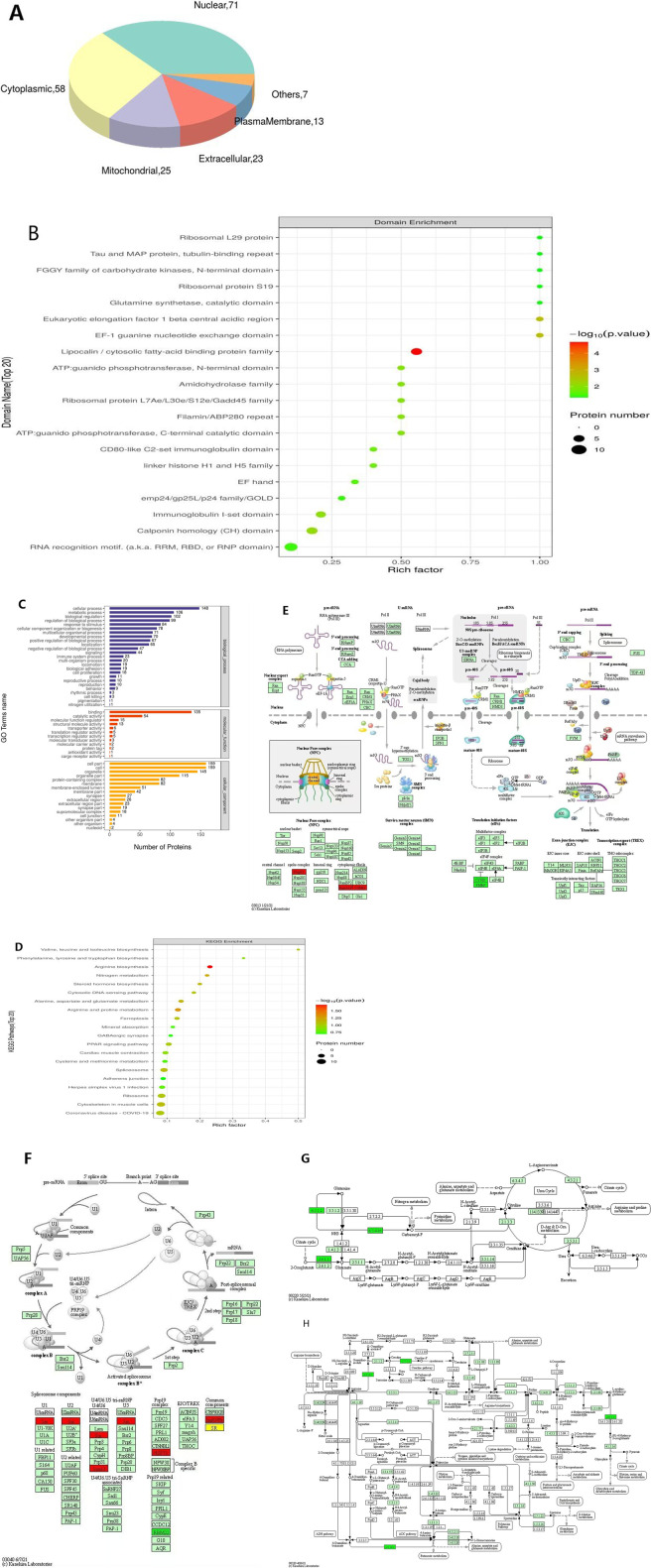
**(A)** Subcellular localisation in normally developing and developmentally delayed groups. **(B)** Domain enrichment analysis (Top 20 domains) in normally developing and developmentally delayed groups. This figure illustrates domain enrichment patterns, with the x-axis representing the enrichment factor (Rich Factor ≤ 1). The enrichment factor was defined as the ratio of DEPs annotated to a given domain relative to the total number of identified proteins annotated to that domain. The size of each bubble corresponds to the number of DEPs within the respective domain category. The colour gradient reflects the statistical significance of domain enrichment, as determined by Fisher’s Exact Test. Specifically, the colour intensity represents the transformed P-value (−log_10_); a deeper red indicates greater significance (smaller P-values). Domain prediction for DEPs was performed using InterProScan software. The full analytical results are provided in supplementary tables, and the protein count per domain (top 20) is additionally visualised as a bar chart. **(C)** GO annotation statistics (Level 2) of DEPs in normally developing and developmentally delayed groups. The figure presents second-level GO (GO Level 2) functional annotations categorised into biological processes (blue), molecular functions (red) and cellular components (orange). The y-axis displays these functional classifications, while the x-axis indicates the corresponding number of DEPs assigned to each category. **(D)** KEGG pathway enrichment analysis (top 20 pathways) of differentially expressed proteins in normally developing and developmentally delayed groups. This figure illustrates pathway enrichment patterns, where the x-axis represents the enrichment factor (Rich Factor ≤ 1), calculated as the ratio between DEPs annotated to a specific KEGG pathway and the total number of identified proteins annotated to that pathway. Bubble size corresponds to the quantity of DEPs within each pathway. Colour intensity reflects the statistical significance of enrichment (Fisher’s Exact Test), with the gradient representing transformed P-values (−log_10_); deeper red indicates greater significance (smaller P-values). **(E)** Upregulated KEGG pathway nucleocytoplasmic transport (mmu03013) in the M-8C vs. C-8C comparison group. The pathway name appears in the upper left corner. Within the diagrammatic representation, red boxes denote upregulated DEPs, green boxes indicate downregulated proteins, and yellow boxes represent pathways involving multiple proteins. Small circles identify small molecule metabolites, while larger boxes represent other related pathways. **(F)** KEGG pathway diagram of spliceosome (mmu03040) in the M-8C vs. C-8C comparison group. **(G)** KEGG pathway diagram of arginine biosynthesis (mmu00220) in the M-8C vs. C-8C comparison group. **(H)** KEGG pathway diagram of arginine and proline metabolism (mmu003300) in the M-8C vs. C-8C comparison group. The pathway name is displayed in the top-left corner of each diagram. Red boxes indicate upregulated DEPs, while green boxes represent downregulated proteins. Yellow boxes identify protein complexes or pathways containing multiple proteins. Small circles correspond to small molecule metabolites, with large ovals depicting connections to other biological pathways.

Bioinformatic analysis of DEPs between the normal development group (C-8C) and delayed development group (M-8C) was performed through subcellular localisation prediction and functional enrichment analysis using GO, KEGG and WikiPathways databases. Subcellular localisation analysis revealed that DEPs were predominantly localised to the nucleus (36.04%), cytoplasm (29.44%), mitochondria (12.69%), extracellular space (11.68%) and plasma membrane (6.6%) (Figure 3A). Domain enrichment analysis of the top 20 enriched domains demonstrated significant involvement in RNA processing and translation, lipid metabolism and signalling, cytoskeletal dynamics, calcium-mediated signal transduction, and fundamental metabolic pathways (Figure 3B). GO enrichment analysis categorised DEPs into cellular components, molecular functions and biological processes. The most enriched cellular components included cell membranes, extracellular regions and organelles. For molecular functions, DEPs were primarily associated with RNA binding and calcium ion homeostasis. Biological process analysis revealed enrichment in metabolic pathways, cell proliferation and signal transduction (Figure 3C). KEGG pathway analysis identified cytoskeletal regulation and spliceosome pathways as the most significantly enriched (Figure 3D). Detailed mapping of DEPs in the spliceosome pathway is presented in Figures 3E,F, while amino acid metabolism mapping is shown in Figures 3G,H. Following FDR correction for multiple comparisons, the statistical support for some results associated with high FRA values was weak and did not reach statistical significance, thus suggesting the relatively limited reliability of these observations.

### 3.4 FABP4 protein verification

**FIGURE 4:**
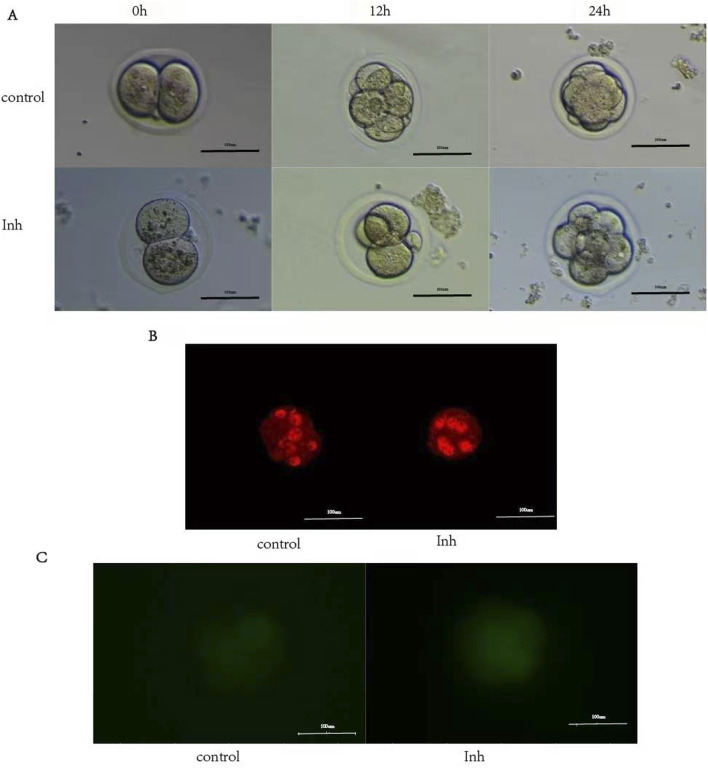
**(A)** The effect of interference treatment on embryonic development at D0, D1, and D2 stages. Representative images of embryos in the control group and interference group at D0, D1, and D2. Scale bar = 100 μm. **(B)** The effect of fetal bovine serum (FBS) on target protein expression in mouse embryos (confocal microscopy). Compared with the control group, the fluorescence intensity of the cell nuclei in the FBS-treated group was significantly reduced, indicating the inhibition of target protein expression. Scale bar = 100 μm. **(C)** The effect of FBS on reactive oxygen species (ROS) levels in mouse embryos. The fluorescence intensity of ROS was significantly increased in the FBS-treated group, indicating elevated oxidative stress. Scale bar = 100 μm.

Next, we investigated the role of FABP4 in embryonic development (Figure 4A). The protein FABP4 exists in early embryos and is mostly localised to the cytoplasm and nucleus, Compared with the control group, the nuclear expression level of the target protein was significantly decreased in the interference group. In addition, there was no significant difference in nucleocytoplasmic distribution among different blastomeres, and the overall protein expression pattern was uniform in embryos (Figure 4B). When FABP4 was inhibited, embryonic development slowed down (Figure 4A); this also led to an increase in the levels of ROS in embryos (Figure 4C).

## 4 Discussion

In this study, we successfully identified several high-abundance proteins, including actin, GAPDH, histone HSP90, and PCNA, in mouse embryos using data-independent acquisition (DIA)-based proteomics. The detection of these proteins not only validates the sensitivity and reliability of our experimental approach but also provides critical insights into the molecular mechanisms governing embryonic development. Actin and GAPDH, encoded by housekeeping genes (Eisenberg and Levanon, 2013), are well-characterised proteins that play key roles in cytoskeletal maintenance and basal metabolic processes (Vigelsø et al., 2015). The consistent detection of these proteins across samples indicates that the experimental workflow, including tissue lysis, enzymatic digestion, and mass spectrometry analysis, had been optimised effectively. The elevated expression of PCNA during the cleavage stage, previously confirmed by immunofluorescence and western blotting (Fan and Li, 2024), further supports the robustness of our dataset. Functional analysis further revealed that the identified proteins are associated with core developmental processes: the prominent expression of ENO1 and LDHA suggests a metabolic shift towards glycolysis, consistent with the adaptation of early embryos to low-oxygen conditions (Bastos et al., 2018). Notably, the complete depletion of histone H3.3 has been demonstrated to cause developmental arrest (Jang et al., 2015), thus highlighting its essential role. With regards to cell polarity establishment, TPM3 contributes to polarisation by regulating the organisation of actin filaments (Vining and Mooney, 2017), while VIM, an intermediate filament protein, maintains mechanical stability in developing embryos (Kelly, 2020). Collectively, these findings demonstrate that DIA-based proteomics effectively captured key molecular signatures associated with metabolic reprogramming and morphogenetic events during murine embryogenesis. This dataset thus serves as a valuable resource for future mechanistic studies and potential applications in reproductive medicine.

Among metabolic regulatory subtypes, Q5FW60 (major urinary protein 20, Mup20), P04117 (fatty acid-binding protein 3, FABP3), and P11404 (apolipoprotein A1, APOA1) [Acetyl (Protein N-term)] were significantly downregulated. Notably, their corresponding peptides contained methylation modifications, DGETFQLMELYGREPDLSSDIKEK (M8), LVSSENFDDYMK (M11), DGDKLVVECVMK (C9), and ADAFVGTWK (Acetyl), potentially influencing lipid transport and energy metabolism (Furuhashi and Hotamisligil, 2008). This downregulation may indicate impaired lipid-mediated energy provision following the exposure of embryos to fetal bovine serum. In contrast, homeostasis-associated subtypes, including Q60590 (LCN2, neutrophil gelatinase-associated lipocalin) and O08716 (retinol-binding protein 4, RBP4), maintained stable expression, likely supporting essential embryonic processes through iron ion homeostasis and vitamin A metabolism regulation (Wan et al., 2024). The observed enrichment in the lipocalin family reflects the increased relative abundance of specific highly expressed subtypes rather than uniform upregulation. Notably, P22935 and A2BIM8 were both expressed at high levels in test samples and met differential expression criteria, substantially contributing to the domain enrichment signal. The downregulation of lipid metabolism-related subtypes may signify a developmental transition from maternal lipid dependence to zygotic autonomous metabolic regulation (Leese, 2012).

GO enrichment analysis revealed significant enrichment of cytoskeleton-related proteins in the test dataset (P < 0.01, FDR = 0.0897). The proportion of these proteins was markedly higher in the interference group (14.55%) than in the control group (5.82%), suggesting that cytoskeletal processes may play a critical role in fetal bovine serum-mediated interference. Of the 24 enriched cytoskeleton-related proteins, several have been implicated in neuronal differentiation and axonal guidance. Specifically, Crmp1 mediates reorganisation of the actin cytoskeleton in response to extracellular signals (Nakamura et al., 2014), while IQGAP1 regulates the dynamics and assembly of the actin cytoskeleton (Wickström et al., 2010). Furthermore, Afadin, an actin filament-binding protein, interacts with nectin and localises to cadherin-based adherens junctions (Ikeda et al., 1999). Data from embryonic samples further suggested that cytoskeletal protein enrichment may indicate developmental delay or arrest.

Enrichment analysis of amino acid metabolism-related pathways demonstrated that arginine biosynthesis (mmu00220) and arginine and proline metabolism (mmu00330) exhibited moderate enrichment in the test dataset. Within the arginine biosynthesis pathway, three associated proteins, specifically P05202 (ornithine aminotransferase) and P15105 (argininosuccinate synthase), were identified in the test group. While this enrichment did not survive rigorous multiple testing correction (P = 0.019, FDR = 0.967), it may nevertheless suggest certain biological characteristics, such as the regulation of metabolic adaptation. The activation of arginine biosynthesis might represent an adaptive response to embryonic nitrogen metabolism requirements or specific cell type demands, particularly those related to rapid proliferation, antioxidant defence, or immune modulation.

With regards to the arginine and proline metabolism pathway (mmu00330), the enrichment of four proteins, including Q6P8J7 (proline dehydrogenase) and Q9D964 (arginase-2), also exhibited borderline significance (P = 0.047, FDR = 0.967), implying possible biological consequences, including the microenvironmental stress response. It is possible that proline metabolism could participate in cellular osmoregulation or provide protection against oxidative stress (Phang, 2019). In embryos exhibiting developmental delay, we observed the significant downregulation of lipocalin family members (including FABP3 and LCN2) and proteins related to the arginine-proline metabolism pathway (mmu00330). The reduced expression of FABP3 (fatty acid-binding protein 3) may directly impair the utilisation of maternal lipid for energy production, thus necessitating a compensatory shift to glucose metabolism. This metabolic shift contradicts the typical early embryonic preference for lipid-based energy sources (Herrmann et al., 2023). Concurrently, the suppression of arginine biosynthesis enzymes (P05202, P15105) may disrupt functionality of the urea cycle and reduce NO signalling (Contreras et al., 2006). These coordinated metabolic alterations likely create an overall imbalance in energy supply, representing a fundamental mechanism underlying developmental delays. The pronounced enrichment of cytoskeletal organisation proteins and S100 calcium-binding proteins indicates abnormal differentiation patterns in developmentally delayed embryos (Mrazkova et al., 2018). Specifically, the impaired calcium-dependent functionality of S100A6 (O54774) could compromise the establishment of cell polarity, thus resulting in defective expansion of the blastocyst cavity. These observations show a strong correlation with protein expression profiles documented in clinical cases of failed embryonic compaction (Li et al., 2015). Marked enrichment in the negative regulation of mRNA splicing (GO:0048025), particularly the downregulation of SR protein family members, may induce aberrant alternative splicing patterns. This could create imbalances in critical embryonic gene isoforms (such as *OCT4* and *SOX2*), ultimately disrupting the equilibrium between the maintenance of pluripotency and the initiation of differentiation (Tadros et al., 2021). These findings suggest that exogenous components in fetal bovine serum (FBS) may perturb the delicate regulatory network controlling zygotic genome activation.

The embryo developmental rate has become a key focus in modern reproductive medicine research. Substantial evidence demonstrates that endogenous regulatory mechanisms exert a profound impact on developmental progression. For example, ovarian stimulation can alter the kinetics of embryonic development (Kurumizaka et al., 2024), critical mitochondrial metabolic factors can govern developmental velocity (Diaz-Cuadros, 2024), and preovulatory follicle diameter is known to correlate significantly with embryonic developmental potential (Clark et al., 2024). With regards to exogenous intervention mechanisms, research has established connections between the serum levels of oxidative stress metabolites during oocyte retrieval and delayed blastocyst development following OPU-ICSI procedures (Hedia et al., 2024). Furthermore, IGF-1 supplementation allows for the targeted modulation of embryonic growth speed (Carrillo-Gonzalez et al., 2024), while the manipulation of temperature in zebrafish models enables the precise control of developmental timing (Urushibata et al., 2021). Clinically significant associations exist between developmental kinetics and outcomes. For example, delayed blastocyst development may contribute to recurrent implantation failure (Ohara et al., 2025), while increased ooplasmic volume accelerates cleavage-stage progression (Murakoshi et al., 2013). In equine species, *in vitro* developmental rates influence both foal survival rates and sex ratios (p < 0.05) (Claes et al., 2020). Current quality control standards for human *in vitro* embryo culture systems predominantly depend on murine embryo assays, in which blastocyst expansion rates generally surpass 80%. Most animal models utilize single-gene knockout/knockdown approaches due to the practical difficulties in obtaining human embryos. Recent developments in DIA-based high-precision proteomic sequencing now facilitate innovative investigations into the early embryonic expression of proteins. The application of this technology for assessing disease-associated embryonic models under multi-pathway regulation provides a valuable foundation for future advancements in human embryonic diagnostics.

Currently, there is limited literature on the role of the FABP4 protein in early embryos. In the present study, we showed that the inhibition of FABP4 can delay embryonic development; however, whether this can also cause changes in the cytoskeleton still needs further investigation. FABP4 is known to be linked to the deposition of fat, and a dark cytoplasm in human embryos is associated with reduced fertility (Xia, 1997). After analysing the transcriptomic results of early embryos at the 2-cell, 4-cell, and 8-cell stages (Yan et al., 2013; Xue et al., 2013; Madissoon et al., 2014), we found that the transcriptomic expression level of *Fabp4* was zero. Furthermore, *Fabp4* expression was not detected in the early embryonic transcriptomes of rhesus monkey (Chitwood et al., 2017), pig (Zhou et al., 2025), and mouse (Sun et al., 2024). Across diverse organisms, the maternal transcriptome undergoes remodeling and complete degradation to ensure normal embryonic development; this occurs even when some maternal transcripts and zygotic transcripts are derived from the same gene (Tora and Vincent, 2021). Once these maternal transcripts have fulfilled their functions, the maternal-to-zygotic transition (MZT) marks the transfer of developmental control from gene products encoded by the maternal genome to those encoded by the zygotic genome. MZT comprises three major events: the clearance of a subset of maternal mRNAs, the initiation of zygotic transcription, and the remodeling of the cell cycle. In each species, MZT occurs at a highly reproducible time point during development, as these three processes are tightly regulated by a series of feedback mechanisms (Brantley and Di Talia, 2024).

Fatty acid binding protein 4 (FABP4) is present in fat, macrophages, liver, limbs, the entire brain, and the placenta, and plays various roles in reproduction, pregnancy, and the health of offspring (Ohguro et al., 2024; Shi et al., 2023b). The most common physiological ligand of FABP4 is unesterified fatty acids, which transport unesterified fatty acids from the cell membrane to different organelles (nucleus and mitochondria) (Amiri et al., 2018). The inhibition or silencing of FABP4 reduces the intracellular levels of free fatty acid, increases the production of ROS, lowers mitochondrial membrane potential, and reduces ATP content (Sun et al., 2026). Fatty acids are not only crucial for the storage of energy substrates but also essential for maintaining cell membranes, as the surface area of the plasma membrane significantly increases during embryonic division. Between the two-cell and four-cell stages, the surface area of the plasma membrane increases by 74%, indicating a greater increase in the late embryonic implantation stage (Pratt and George, 1989). The fat content in embryos is known to significantly decrease during development (Romek et al., 2009). We found that the inhibition of *Fabp4* leads to insufficient cellular energy supply and slows down the formation of the cell membrane.

**FIGURE 5:**
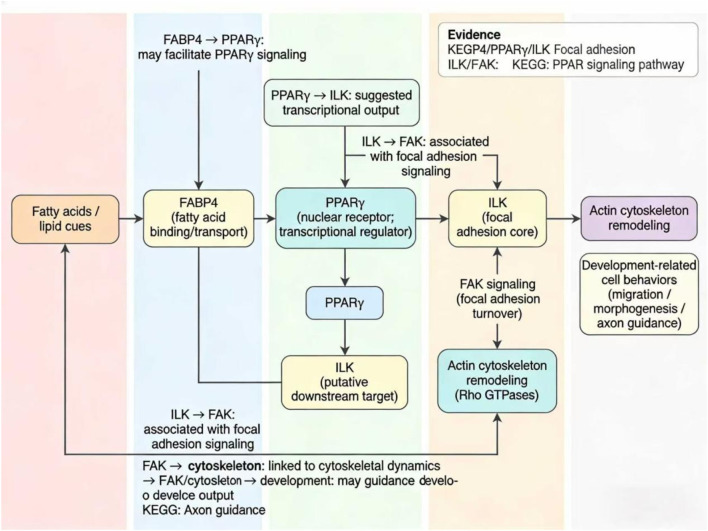
Schematic diagram of the putative mechanism underlying the Fabp4-mediated signaling axis during early embryonic development.

Previous studies of FABPs and peroxisome proliferator-activated receptors (PPARs) revealed that A-FABP (FABP4) can specifically bind and activate PPARγ. Under ligand stimulation, FABP4 moves from the cytoplasm to the nucleus and effectively transmits the ligand to PPARγ through direct protein-protein interactions, thereby enhancing its transcriptional activity. This selective regulation is highly specific and is particularly important under low ligand concentrations, playing a crucial role in maintaining the normal function of PPARγ (Casagrande et al., 2025). The results of nuclear magnetic resonance and crystallography further indicate that the hydrophobic lock residue phenylalanine (Boss et al., 2015) in human FABP4 is the main driving factor for combining ligand characteristics with biological outcomes (Tan et al., 2002), and that the inhibition of FABP4 can inhibit the activity of PPARγ (Wirth et al., 2022). After treating Panu 02 cells with 100 ng/mL of FABP4 for 48 h, the generation of ROS was also observed to decrease. The exogenous addition of 100 ng/mL of FABP4 was shown to increase the proliferation of Panc-1 cells, while 20 and 40 ng/mL did not (Ren et al., 2025). In two early PPAR-γ inhibition experiments on bovine embryos, one experiment used siRNA to inhibit expression but failed to identify any obvious cell changes (Pérez-Gómez et al., 2024); however, different results were acquired following the use of inhibitors, with a reduction in cell number and reduced embryo implantation ability (McGraw et al., 2024) These observations explained the remodelling of PPARγ transcription in early embryonic development and how this regulates both cell proliferation and invasion. The inhibition of PPARγ can cause dual stasis of the G and M stages of the cell cycle; following the inhibition of PPARγ, microtubule loss is caused by the post-transcriptional regulation of tubulin (Schaefer et al., 2007). Based on the above analysis, we constructed a schematic diagram of the Fabp4-mediated signaling axis underlying the putative mechanism during early embryonic development as shown in Figure 5.

## 5 Conclusion

Fetal bovine serum-induced embryonic developmental delay may disrupt normal embryogenesis by interfering with the dynamic coupling network that coordinates cytoskeletal organization, nuclear transport, and metabolic processes, ultimately resulting in delayed developmental phenotypes. This developmental asynchrony may lead to a temporal mismatch with the endometrial implantation window, potentially contributing to pregnancy failure. In our research, we demonstrated that the fatty acid transporter FABP4 exists in early 8-cell mouse embryos and that the *Fabp4* inhibitor BMS-309403, at a concentration of 200 nM, can cause embryonic retardation and increase the level of ROS.

## Data Availability

The mass spectrometry proteomics data have been deposited to the ProteomeXchange Consortium (https://proteomecentral.proteomexchange.org) via the iProX partner repository [Ma et al., 2019; Chen et al., 2021] with the dataset identifier PXD079804.
